# An Empirical Analysis of the Current Need for Teleneuromedical Care in German Hospitals without Neurology Departments

**DOI:** 10.1155/2010/916868

**Published:** 2010-06-29

**Authors:** G. W. Ickenstein, S. Groß, D. Tenckhoff, P. Hausn, U. Becker, J. Klisch, S. Isenmann

**Affiliations:** ^1^Department of Neurology and Stroke Unit, HELIOS General Hospital Aue, Technical University Dresden, Gartenstr. 6 - 08280 Aue, Germany; ^2^Department of Neurology, Carl-Gustav Carus University Hospital, 2-Fetcherstr. 74, 01307 Dresden, Germany; ^3^Department of Neuroradiology, HELIOS General Hospital, 3-Nordhäuserstr. 74, 99089 Erfurt, Germany; ^4^Department of Neurology, HELIOS General Hospital Wuppertal, Witten-Herdecke University, 4-Heusnerstr. 40, 42283 Wuppertal, Germany

## Abstract

*Indroduction*. At present, modern telemedicine methods are being introduced, that may contribute to reducing lack of qualified stroke patient care, particularly in less populated regions. With the help of video conferencing systems, a so-called neuromedical teleconsultation is carried out. *Methods*. The study included a multicentered, completely standardized survey of physicians in hospitals by means of a computerized on-line questionnaire. Descriptive statistical methods were used for data analysis. *Results*. 119 acute hospitals without neurology departments were included in the study. The most important reasons for participating in a teleneuromedical network is seen as the improvement in the quality of treatment (82%), the ability to avoid unnecessary patient transport (76%), easier and faster access to stroke expertise (72%) as well as better competitiveness among medical services (67%). The most significant problem areas are the financing system of teleneuromedicine with regard to the acquisition costs of the technical equipment (43%) and the compensation for the stroke-unit center with the specialists' consultation service (31%) as well as legal aspects of teleneuromedicine (27%). *Conclusions*. This investigation showed that there is a high acceptance for teleneuromedicine among co-operating hospitals. However these facilities have goals in addition to improved quality in stroke treatment. Therefore the use of teleneuromedicine must be also associated with long term incentives for the overall health care system, particularly since the implementation of a teleneuromedicine network system is time consuming and associated with high implementation costs.

## 1. Introduction

Stroke patients should be medically treated in specially designed facilities, so-called stroke units, because of the high efficacy of the care provided there [1] and recommended by the “European Stroke Initiative (EUSI)” as well as that of the “German Stroke Society (DSG)” [2]. In Germany, a total of 195 stroke units has been set up so far, enough to provide care for approximately half of all stroke patients in Germany. An increase in the number of stroke units to 250 has been planned in order to bring this effective care to almost 85% of stroke patients on the long-term [3]. However, acute care will continue to be provided in hospitals without stroke units in the future, especially in rural areas, where economic and staff limitations prevent the establishment of specialized neurological stroke units [4]. 

The question then arises, whether the use of modern teleneuromedicine methods could contribute to reducing the deficit in stroke patient care, particularly in less populated regions. In these areas, which lack neurological expertise, acute medical treatment of stroke patients usually occurs in the internal medicine department [5, 6]. Within the context of teleneuroconsultation, discussion and deliberation between two or more doctors regarding the best diagnostic and therapeutic approach for the acute stroke patient can take place. In teleneuromedical settings, the stroke expert is connected by video and sound transmission, observing the examination of the patient, which is carried out by the doctor at the regional hospital [7].

**Figure 1:**
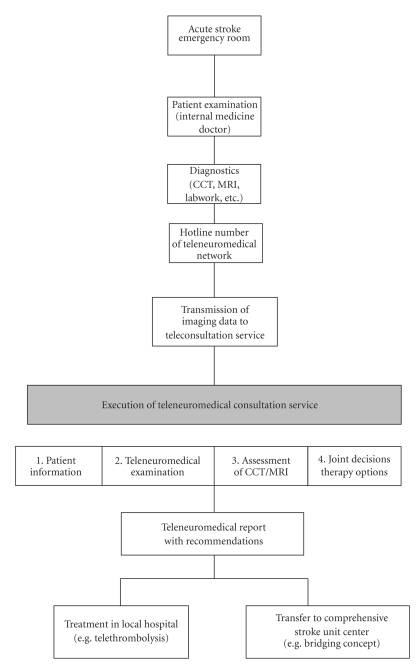
Sequence of events in teleconsultations in a teleneuromedical network [8, 9].

In addition, the radiological image data (CT or MRI) collected in the regional hospital is electronically transmitted to a server platform that can be accessed by the stroke expert. On the basis of this information as well as the clinical impressions that the stroke expert receives during the video conference, the remote diagnosis and related therapeutic instructions or recommendations are determined and communicated to the doctor with a datasafe consultation sheet (see Figure 1) [8]. The study considers to what extent hospitals in Germany have already fulfilled the requirements for participation in a telehealth care network and to what extent these methods are already in use in Germany. Furthermore, an empirical analysis evaluates the advantages and disadvantages of teleneuromedicine. Finally, the findings of the investigation are discussed in relation to existing teleneuromedical networks and questions are answered as to what demands these special networks can meet in the future.

## 2. Methods

The study included a multicentered, completely standardized survey of physicians in hospitals by means of a computerized online questionnaire. Those selected for the investigation received an E-mail letter inviting them to participate. It contained information regarding the purpose and goal of the empirical analysis. It also included a link to an internet site where participants could fill out the questionnaire. In the analysis of the data, descriptive statistical methods were used. Both univariate (bar graphs and pie charts) and bivariate (crosstabulation) methods were used in the analysis according to GCP guidelines (EK 255102007). 

Of a total of 2,104 hospitals in Germany, university hospitals, specialized hospitals and facilities with stroke units in a neurology department were excluded. Information regarding each hospital equipment and configuration was taken from their internet sites. This selection reduced the total by 845 to be 1,259 hospitals, constituting the basic population of the survey. However, not all of them could be reached on-line, since in 494 cases the hospital e-mail address was not available. Therefore, a total of 765 hospitals were surveyed (61%) and 346 (45%) hospitals could be directly reached with a general address in the hospital (i.e., info@...). Out of the 765 hospitals that fulfilled the inclusion criteria, 134 hospitals entered the website and completed the survey, amounting to a return rate of 18%. Out of the 134 participating hospitals, 15 had in addition to an internal medicine also a newly opened neurology department. These facilities also did not meet the inclusion criteria for the empirical analysis and were not included in the final analysis. The following descriptions and evaluations thus refer to 119 acute hospitals without any neurology department. 

## 3. Results

**Figure 2:**
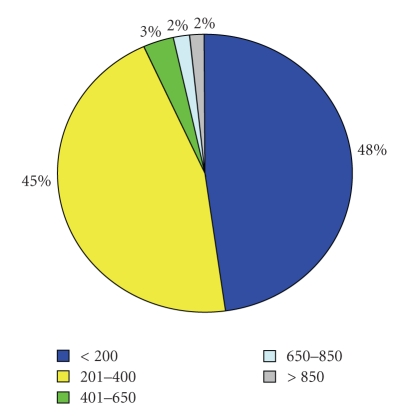
Size of hospitals according to number of beds.

As shown in Figure 2 predominantly hospitals with less than 200 beds (48%) or between 200–400 beds (45%) in small towns (5,000–100,000 inhabitants) took part in the survey (80%). 15% of the remaining participants belonged to the category “big city” (>100,000 inhabitants) and 5% to “rural regions” (<5,000 inhabitants). Regarding the current status of teleneuromedicine in hospitals without neurology departments, 36% of the responding hospitals are already connected to a teleneuromedical network and 14% of the participants are in negotiations with a stroke unit, whereas 30% plan to become active in telemedicine stroke treatment in the future. For only 11% of the participating hospitals telemedicine does not seem an option, while 8% did not answer this question. 

**Figure 3:**
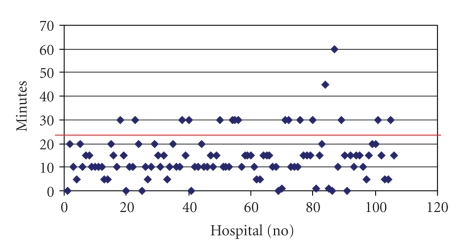
“Time to CT” for each hospital with a cut-off time at 25 minutes.

The availability of an hospital network with high transmission speed is queried. In addition to these specific technical requirements, certain organizational conditions must be fulfilled in order for a hospital to take part in a teleneuromedical program. According to recommendations by the German Neurological Society (DGN) the time period from the arrival of the patient in the emergency room of the cooperating hospital to the beginning of the diagnostic phase should be no longer than 25 minutes (“Time to CT”). Therefore the distance between the emergency room and the location of the imaging center within the cooperating hospital is an important factor. Besides fast and easy access it is also required that the CT can be performed at any time 24 hours/7 days. The arithmetic mean of the responses “Time to CT” was 14.42 minutes with a median of 12.50 minutes and a standard deviation of 9.95 minutes. 17 participants (16%) could not comply with the required time period (see Figure 3).

In 114 of the participating hospitals (96%), the performance of a CT is possible, only 4% cannot meet this requirement. The DICOM interface needed for the CT image server exchange constitutes a critical component. 82% (94/119) reported that their CT meet this requirement but different possibilities for remote data transmission are used (see Table 1). Networking connectivity was possible in 85% (101/119) of the hospitals with different transmission speeds performed: <100 MB/s in 3%, 100 MB/s in 28%, 1 GB/s in 7%, >1 GB/s in 8%, and answer “not sure but >100 MB/s” in 39%. 

In case of complications (e.g., malignant brain edema or intracerebral bleeding) associated with thrombolytic therapy, the transfer of a stroke patient to a neurosurgery department may be necessary. Moreover, for bridging concepts in individual cases it is expedient to transfer the patient to an comprehensive stroke unit center for specialized neuroradiology interventions [9]. According to the code “Other complex neurological treatments of acute stroke (G-DRG OPS 8-98b)” such a facility should be reachable within 30 minutes. This logistic requirement is met by 71% of the participants. Concerning early rehabilitation measures, 117 participants (98%) responded that they have the capacity to carry out early physiotherapy. Speech and language therapy is available to only 71% of the hospitals, and occupational therapy to 59%. 

The most important reason for participating in a teleneuromedical network is the improvement in treatment quality (82%) and the ability to avoid unnecessary patient transport (76%). Easier and faster access to expertise (72%) and the related improvement in establishing a final diagnosis (66%) constitute further medical arguments. Furthermore, participants had to assess whether the use of teleneuromedicine increased the competitiveness of their hospital, to which 67% responded in the affirmative. Table 2 shows that mainly small hospitals with <200 beds voted for this statement while hospitals with >200 beds do not have a clear tendency. 

**Figure 4:**
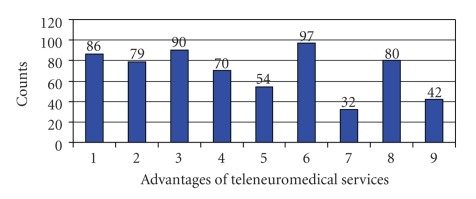
Advantages of teleneuromedical services: 1. easier and faster access to expertise, 2. improvement in establishing a diagnosis, 3. avoid unnecessary patient transport; 4. ability to carry out i.v. thrombolysis, 5. educational development of doctors, 6. improvement in the quality of treatment, 7. reduction of length of stay, 8. better competitiveness, 9. increasing patient numbers.

The ability to carry out systemic (i.v.) thrombolysis within their own hospitals was considered by 59% of participants and the improved professional training of doctors within the hospital was also seen as a possible motive. The reduction of length of hospital stay (LOS) in stroke patients was not associated with a main incentive for the cooperating hospitals (see Figure 4). 

**Figure 5:**
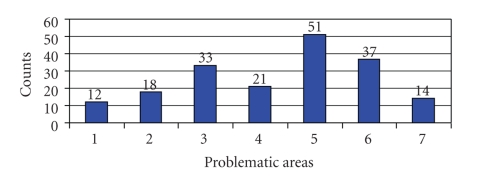
Problematic areas of teleneuromedical services: 1. acceptance of teleneuromedicine 2. conflict with competency boundaries, 3. legal aspects, 4. data security reasons, 5. financing problems, 6. compensation for consultation service, 7. acquisition costs for technical equipment.

The most significant problem area for the hospitals seems to be the financing system of telemedicine with regard to the acquisition costs of the technical equipment. This aspect represents the main factor in the modal value of the answers (43%). In addition to this the compensation for the stroke-unit center with the specialist's consultation service (31%) as well as the legal aspects of teleneuromedicine (27%) present a risk. However, data security reasons (18%), potential conflict between different specialties with competency boundaries (15%) as well as operating costs (12%) are deemed much less significant. The least problematic aspect according to the hospitals is the acceptance of teleneuromedicine among doctors (10%). 90% of the participating hospitals accept teleneuromedicine as a good supplement to the well established stroke care (see Figure 5).

## 4. Discussion of the Empirical Analysis

With stroke becoming increasingly even more important and neurological stroke units lacking in rural areas, teleneuromedicine emerges as a promising technique in stroke care. The present work focuses on quality improvement with the help of a teleneuromedical network system. The advantages of teleneuromedicine in acute stroke include faster and more accurate diagnoses in participating hospitals, since they have easier access to neurological expertise. To reach this goal it is, however, also necessary that such a health care configuration be accepted by participating doctors and that these doctors in turn be accurately advised. It is possible that doctors in cooperating hospitals may not always be prepared to submit to the competencies of colleagues in the treatment of “their” stroke patients. A negative attitude toward the use of teleneuromedicine might be related to this aspect. However, this was not confirmed by the present investigation. Most participants showed a ready acceptance of teleneuromedicine and ranked possible conflicts arising from competency boundaries very low. 

Interestingly, improved competitiveness was cited as a predominant argument for teleneuromedicine by hospitals with less than 200 beds. This aspect can be explained by various considerations. For example, changes in the hospital system could also affect a facility's catchment area. If a neighboring hospital expands, its attractiveness increases and consequently its catchment area as well, to the detriment of other hospitals, which must adjust to a reduced load. Thus, adjustments to the range of services and capacity are necessary, the more so as with reduced patient numbers high fixed costs can no longer be covered. The result will be a decline in the attractiveness of the facility, bringing with it a further reduction of the catchment area. This process can eventually lead to the closing of a hospital. This process is already ongoing in Germany at present, and will continue into the next few years. Such adjustment mechanisms or focusing on just a few care providers may even be a political aspiration. Conversely, when a hospital, especially a small one, connects to a teleneuromedicine network, thereby improving stroke care and treatment results, its attractiveness among regional care providers will be increased. This hospital would thus have a good argument for justifying its health care mandate and for securing its existence. On the other hand, the competitive advantage can become a direct competition. Assuming each of two neighboring general hospitals, A and B, with very similar services and equipment, care for stroke patients in their respective catchment areas. Hospital A connects to a teleneuromedicine network and can then possibly attract additional stroke patients from the catchment area of hospital B, as a result of the advantages of teleneuromedicine. The precondition is that the rescue service is informed about the teleneuromedical capabilities of hospital A and delivers stroke patients primarily to this facility. Additional billable codes result for hospital A, which are tied to additional revenue [11]. However, at the same time further direct treatment costs arise from the expansion of diagnostics and treatments for the additional patients. Furthermore, one might ask if hospital A has sufficient capacity for the increased patient numbers; although if, as described, the length of in-hospital stay is reduced by the use of teleneuromedicine, it may be possible to care for more patients without increasing the hospital's capacity [7, 12]. It must be noted, however, that the reduction of length of stay was largely not confirmed by the data provided by participating hospitals in this study. 

As was to be expected, financing of teleneuromedicine currently constitutes the most significant problem area. However, this aspect is not very prominent in hospitals already active in teleneuromedicine (14%). These hospitals are mainly located in Bavaria, where they are often financially supported by the state or health insurances (e.g., TEMPIS or STENO) [13]. But facilities which are in negotiations with network partners or plan to become active in teleneuromedicine might see a substantial, negotiable factor between the involved parties. Especially in those facilities for which a connection to a teleneuromedicine network is out of the question the problem of financing is quite prominent. Considering the necessary investments (hardware, software, IT support, broadband etc.), lack of sufficient financial resources can be a significant reason for the failure of teleneuromedicine in a cooperating hospital. This problem can be discussed with health insurers and the state since both can profit from the use of teleneuromedicine. As a result of quality improvement in stroke care throughout the state this sort of health care configuration in less developed regions can reduce the number of those needing long-term care and the direct and indirect costs associated with it [14]. Besides this, savings can be achieved through the elimination of unnecessary patient transport. The problem is that, these effects are not immediately evident and are only noticed by the cooperating hospitals in the long term. Which operating network costs or savings result concretely are not as easily enumerated on basic acquisition. The consequence of this lack of internalization and the associated lack of incentive could be a reason that the use of teleneuromedicine is still partly out of the question for a given facility. If a list of advantages and problems is composed, however, the advantages will prevail. It should therefore be mentioned that the opportunities provided by teleneuromedicine are ranked higher by the hospitals taking part in the survey than the risks connected with it. A generalization of this statement, however, cannot be made offhand. In the discussion of the survey results, it must be kept in mind that only a certain percentage of the total population of all acute hospitals in Germany responded and that those hospitals are particularly interested in teleneuromedicine. Hospitals for which the use of telemedicine in stroke care is out of the question tend, perhaps, not to answer. 

Moreover, aspects related to the implementation of thrombolysis therapy are important. A large portion of the facilities can in principle envision performing thrombolysis with the help of a teleneuromedically connected stroke expert. Doctors in cooperating hospitals are prepared to trust their colleagues from the connected stroke unit and to rely on their evaluation. While the doctors in the cooperating hospitals bear the entire responsibility for thrombolysis treatment, for most of them the medical advantages and the associated benefits to the stroke patients are certainly in the foreground, so that when weighing the options, the arguments in favor of a thrombolysis treatment or bridging concept win out. The economic aspect of reducing unnecessary transfers, which are often very time consuming, can be largely confirmed by participating hospitals. If a stroke patient does not reach the cooperating hospital within 4.5 hours, after onset of symptoms [15], intravenous thrombolysis is no longer justified. Therefore, time should be used as effectively as possible, since up to the point of administration of the medication, appropriate examinations as well as the teleconsultation must be completed, and the success rate of thrombolysis decreases with each passing minute [16]. The brevity of this time period and especially the lengthy pre-hospital time periods are the most significant reasons for low thrombolysis rates in cooperating hospitals. Given the logical and literature-supported supposition that time-to-recanalization is crucial, rapid and safe recanalization is a primary goal. The initial teleconsultation with CT and CT angiographic findings can quickly determine whether the patient is a suitable candidate for an interventional neuroradiological procedure, although—up to now the ideal method by which a rapid and safe recanalization is achieved is not clear. In addition to limited recanalization rates, current IA therapies, particularly IA thrombolytics and mechanical devices, can take hours to achieve recanalization. In this case a so-called bridging procedure with initially intravenous thrombolysis can first be begun, and the patient can then be transferred to a specialized interventional neuroradiology while undergoing treatment without time delay [9, 17–19]. In acute basilar artery occlusion, M1 occlusion of the middle cerebral artery and occlusion of the internal carotid artery, intra-arterial thrombolysis or/and endovascular mechanical recanalization may result in higher recanalization rates than intravenous thrombolysis alone. Bridging IV/IA thrombolytic therapy for such acute stroke patients appears to be safe and yields higher recanalization and improved survival rates, as well as an overall improved chance for a better outcome. However many patients are admitted to community hospitals, where endovascular therapy is usually not readily available. In this setting a teleneuromedical supported proper selection of stroke patients is mandatory, who will benefit from an initialization of thrombolysis within a community hospital with simultaneous referral to a comprehensive stroke center, thus leading to a better functional outcome of stroke patients. However randomized controlled trials will have to confirm the expected benefit of bridging IV/IA thrombolysis with subsequent on-demand mechanical recanalization on clinical outcome [20–22]. 

The present investigation showed that most participants had sufficient equipment to perform thrombolysis. The recommendation of the German Stroke Society, that stroke patients undergo a CT scan within 25 minutes at most, was fulfilled with an average of 14.42 minutes of all responses. Even with a standard deviation of approx. 10 minutes it is still possible to reach this goal. It can be concluded that the internal structures and procedures within most of the participating hospitals seem to be efficient enough to guarantee quick access to a CT scan [23]. In addition, the response patterns show that the technical requirements do not stand in the way of becoming active in teleneuromedical care. The question arises whether in those hospitals which do not meet this requirement, inefficient internal organization could be the culprit. Another reason might be that these hospitals share the use of a CT with other facilities, for example, an external radiology service, and therefore delays may occur. Because of limited availability and the resulting longer “Time to MRI”, it can be deduced that in hospitals having both imaging methods at their disposal, CT will often be primarily used for initial diagnosis in the treatment of stroke patients. Both methods may be used later in the course of the treatment to strengthen the validity of the diagnostics [24, 25]. Failure to meet the 4.5 hour deadline, after which intravenous thrombolysis cannot be initiated, might also be due to an information deficit on the patient's part. For some, the symptoms of stroke are not known, at least not sufficiently so that calling the ambulance is often too late. For this reason there is a continuous necessity to educate the population to the importance of seeking stroke treatment as quickly as possible. It remains questionable to what extent a cooperating hospital can contribute to this, particularly as only one third of the facilities consider this a way to raise the thrombolysis rate. The cooperating hospitals consider the medical exclusion criteria much more significant for the low thrombolysis rate [4, 6, 26]. 

## 5. Examples of Exsisting Telestroke Networks in Routine Practise

Telemedicine technologies have been shown to be useful and effective in the remote neurological evaluation and treatment of acute stroke patients and is now used at several hospitals in Europe and the United States as an option for stroke patients to have access to cerebrovascular expertise. The effect of this concept was evaluated in the TEMPIS project, where five regional hospitals with a telestroke concept were matched with five regional hospitals without a telenetworking system. During two years stroke patients were monitored and the three-month outcome was studied. In an multivariate analysis the stroke treatment in the TEMPIS project showed a significantly better result compared with the nonnetworking hospitals and the thrombolysis rate was ten times higher [13].

Many physicians, especially nonneurologists, remain hesitant to use rt-PA in acute stroke patients, suggesting that additional training methods and tools are desperately needed in many communities [27]. Since the NINDS-sponsored trial of rt-PA in acute stroke was conducted at a relatively small number of experienced stroke centers, one commonly expressed concern is that similar results might not be obtained when rt-PA is used in a variety of clinical settings. After publication of the NINDS trial results more than a dozen reports of experience with rt-PA in open-label, routine clinical use have been published. In 2639 treated patients, the symptomatic intracerebral hemorrhage rate was 5.2% (95% confidence interval 4.3–6.0) slightly lower than 6.4% rate of the NINDS trial (National Institute of Neurological Disorders and Stroke) [28]. The mean total death rate (13.4%) and proportion of subjects achieving a very favorable outcome (37.1%) were comparable to the NINDS trial results [29]. As a result community hospitals will increasingly face medicolegal risks both for treating and for not treating patients with newly available agents. With a “back up” of stroke experts in a professional telenetworking system patients and family members can be assured that they speak with the expert “face to face” or “online” in the emergency unit and that all treatment options are standardized and discussed. This will release a huge burden from the less stroke experienced doctors in the local hospitals in rural areas since a major problem confronting all community hospital stroke programs is one that has been called the “frequency factor”. Since a small number of stroke patients will qualify for acute interventions such as TPA thrombolysis, a stroke team could have difficulties in running effective. A study investigating the routine use of systemic TPA thrombolysis reports an increase in in-hospital mortality after administration of TPA in hospitals with <5 thrombolytic therapies within 1 year [30]. These findings underline the need to have an experienced stroke expert involved in the management of an acute stroke patient since urgent therapeutic decisions in emergency stroke care have to be made on the basis of brain imaging and a structured clinical examination. With a good knowledge of functional and vascular cerebral anatomy the stroke expert can quickly determine the neuroanatomic localization of the brain lesion and can guide special treatment options. Since such experience and resources are available mainly in stroke centers of teaching hospitals a networking system can allow each hospital to have access to the experience of all programs in the network.

In Germany telemedicine is beginning to be further established. For example, the NeuroNet constitutes an implementation of teleneuromedicine in clinical practice and represents an intraregional teleneuromedicine network of hospitals, wherein only hospitals belonging to the HELIOS hospital group take part. This project has been in operation since 2006 [9, 31] counting among its hospitals five certified stroke unit centers, who serve as “providers” of teleneuromedical expertise including neurologist, neurosurgeons and neuroradiologists. These stroke unit centers rotate their on-call services weekly whereas the cooperating hospitals make up the regional facilities or the consumers of teleneuromedical expertise [32, 33]. In NeuroNet (http://www.helios-neuronet.de) the implementation costs were covered by the hospital group and every cooperating hospital has a mobile workstation (VIMED TELEDOC 2) with a video conferencing system, and the consult is processed by a central server that can send and receive digital radiological images in DICOM format [10]. A great advantage is that the mobility of this workstation allows for its use regardless of the location of the consulting physician. Thus, patients in various parts of the hospital can be evaluated. Since every minute lost when dealing with acute stroke implies a loss of viable brain tissue, the network system strives to carry out thrombolysis on site or, with a bridging concept, to start it on site followed by transport to a comprehensive stroke unit center after the diagnosis and initiation of therapy. These networks should help to introduce these bridging concepts in a systematic way. Part of the whole concept is also supervision and quality management with standard operation procedures (SOP) to further develop the network. Adjusted to suit available regional configurations, these networks are intended to lead to a uniform optimization of care for stroke patients. Therefore training of stroke doctors, stroke nurses and therapists are continued and training equipment like the Stroke Lysis Box (IPC:A61F-17/00, patent number 200 09 172.7) and a NIHSS training DVD (http://www.physiothek.com) were indroduced. Within a systematic peer review process quality data from all hospitals are continuously evaluated and routine data with mortality rates are observed over the years to monitor the improvements in quality of stroke care.

Furthermore, the Society of Hospitals in Saxony (KGS), together with the Regional Association of Health Care Providers (LVSK) and the Saxon Ministry of Social Welfare (SMS), have after almost two years of negotiations with all involved parties agreed on a financial framework for improving telestroke care uniformely throughout Saxony, especially in rural areas, through the establishment of teleneuromedicine networks. The comprehensive stroke unit centers are the University Hospital Dresden for eastern Saxony (SOS-NET, http://www.neuro.med.tu-dresden.de/sos-net/) and for southwestern Saxony a trio of comprehensive stroke unit centers with Aue, Chemnitz and Zwickau (TNS-NET). The criteria for the participation of hospitals is clearly defined and a team from the stroke unit centers visits the potential cooperating hospital in order to check the quality criteria. It also instructs doctors, nurses and therapists particulary in operating the equipment and evaluating stroke patients with certain stroke scales. In these networks advanced and professional training is ongoing, and especially nursing staff is educated in a “Stroke Nurse Training Program” lasting six months (http://www.dsg-info.de/pdf/Pflegefortbildung_Helios_Akademie.pdf). The German Stroke Society (DSG) and the German Neurological Society (DGN) recently published standards for telestroke services that will now lead to a general certification of these networks. 

But this safe and effective telestroke and “tele-thrombolysis” service with experienced stroke experts for stroke management requires a 24 hour on demand teleconsultation service that needs to be reimbursed. In TEMPiS, the expenses for this service account 300,000 *€* per year. Based on the calculated savings of subsequent costs by each thrombolysis between 3300 to 4200 *€* [34] the absolute increase of systemic thrombolysis of 75 TPA treatments within one year would result in a total saving between 250,800 and 319,200 *€*. Therefore the teleconsultation service results to be cost-efficient regarding only the consultations of possible thrombolyses. In Denmark the budgetary impact and cost-effectiveness of the national use of thrombolysis for stroke administered via telemedicine was estimated. The result demonstrated that thrombolysis by telestroke network was dominant to conservative management [35]. The incremental cost-effectiveness ratio was calculated to be approximately 50.000 $ when taking a short time perspective (1 year) but thrombolysis was both cheaper and more effective after 2 years and cost effectiveness improved over longer time scales. However studies conducted from a societal perspective compared with those conducted from an institutional perspective have a tendency to overestimate the total revenue. In the absence of ongoing government grant support, any telestroke sponsoring institution must devise a business model that produces a self-sustaining profitable break-even program. The health economic model computations suggest that the macroeconomics costs may balance with savings in care and rehabilitation after as little as 2 years and that potentially large long-term savings are associated with thrombolysis devilered by teleneuromedicine.

In the United States several telestroke projects are established mostly by governmental grants and published in journals (Partners Telestroke Center; STARR; STRokE DOC; REACH; RUN-Stroke; clinicaltrials.gov) [36]. Interestingly the specialist on call (SOC, http://www.specialistoncall.com) project is a privat business model that operates with 15 neurologists covering 65 hospitals in six states dealing with 3.600 teleconsultations per year. The SOC offers flat rates for hospitals where the hospital pays one time 30.000 *€* for the technical equipment and a monthly fee that is adjusted to the size of the hospital. The stroke neurologists are hired by SOC and monthly payed for their stroke expert teleneuromedicine service. However in Germany the teleprojects are always connected to comprehensive stroke unit centers and mostly supported by the state system. In Saxony the costs for the technical equipment of a stroke unit center and a cooperating hospital are covered by the Saxon Ministery of Social Welfare so that the implementation of the network infrastructure was possible for each hospital that could fulfill the standardized inclusion criteria for the statewide telestroke network configuration (30.000 *€* one time payment for each hospital). Together with the health insurance companies in Saxony it was possible to discuss a model, where each teleconsultation can be billed with 1/3 receiving the cooperating hospital and 2/3 receiving the stroke unit center. With this billing model per teleconsult all costs have to be covered including broadband costs, IT specialists, savings for new equipment, and of course payments for the stroke experts and teaching costs. The state concept allows a stable routine teleconsultation service with incentives to use the system and possibilities for growth in order to improve the quality of the telenetworking system. Since an European consensus statement has set the goal of having all persons with acute stroke admitted to specialized treatment facilities [37] establishing such a teleneuromedicine networking system in nonurban areas might be the solution to the difficult reimbursement situation of insurance companies and the problems in finding enough stroke neurologists. 

## 6. Conclusions

In summary, broad implementation of thrombolysis in stroke is supported by the expanding telestroke networks. With the use of modern teleneuromedicine network systems quicker access to neuromedical expertise is possible. Especially the fact that comprehensive coverage by stroke units throughout Germany does not appear to be feasible at present, teleneuromedicine represents a valueable supplement to the existing system of stroke care. However, the use of teleneuromedicine alone cannot replace optimal care provided by comprehensive stroke units. Overall, the hospitals in a network must upgrade and optimize their internal structures in a holistic operational process to improve stroke care but project data show that telestroke and telethrombolysis are practicable and can contribute to the improvement of stroke care in rural hospitals that are too distant from a specialized stroke unit. 

This investigation shows that there is good acceptance for teleneuromedicine among networking hospitals and that doctors see the opportunities offered by this sort of health care configuration. Technical and organizational requirements must be fulfilled in the hospitals and medical product guidelines should be followed along with a stable financing system and the possibility for deductions by the insurers so that even small hospitals can participate in this health care configuration. From a socio-economic point of view it is precisely these facilities in rural areas far away from stroke unit centers that can profit most, in terms of telestroke care. Patient benefits and resulting potentials for cost savings have to be addressed in the teleprojects and in discussion with health care structures as can be seen in Saxony. 

## Figures and Tables

**Table 1:** Technical possibilities for remote data transmission in networking hospitals [10].

Network access	Transmission speed	Suitability for teleneuromedicine	Costs
ISDN (point-to-point)	64–128 KB and more	(i) Nationwide, also available in rural areas	24 euro per month and 128 KB
		(ii) Relatively slow	
		(iii) Only for small image transfers	
		(iv) Ability to bundle cables	

ADSL (Internet)	128–768 KB (upload) 1–16 MB (download)	(i) Fast downloading	20–150 euro per month including connection costs
		(ii) Not available everywhere	
		(iii) Home connection	

SDSL (Internet)	512 KB to 2 MB (upload and download)	(i) Fast Up- and downloading	120–180 euro per month including connection costs
		(ii) Not available everywhere	
		(iii) Suitable for small and mid-sized hospitals	

Satellite (Internet)	1,5 to 24 MB (download no upload)	(i) No upload → ISDN as feedback channel (upload) required	30–130 euro per month according to provider and rate
		(ii) Available everywhere	
		(iii) Badly suited for synchronous connection because of long latency periods	

Leased line SFV (point-to-point)	640 KB to 155 MB (upload and download)	(i) Highest reliability	300–1200 euro per month
		(ii) Possible high speed	
		(iii) Recommended for big hospitals	

**Table 2:** Competitive advantage in relation to the size of the hospital.

*n* = 119 hospitals	Teleneurology provides a competitive advantage	Teleneurology does *not* provide a competitive advantage
Hospitals with ≤200 beds (*n* = 57)	48 (84%)	9 (16%)

Hospitals with >200 beds (*n* = 62)	32 (52%)	30 (48%)

all hospitals	80 (67%)	39 (33%)
